# Supplementary material for: Entangled-photon decision maker
Source: Sci Rep. 2019 Aug 22;9:12229. doi: 10.1038/s41598-019-48647-7 (PMC6706396; doi:10.1038/s41598-019-48647-7)
Supplement: Supplementary file 1 — Supplementary Information: Entangled-photon decision maker [file 41598_2019_48647_MOESM1_ESM.pdf]

Supplementary Information:

Entangled-photon decision maker

Nicolas Chauvet[1,2*], David Jegouso[1], Benoît Boulanger[1],

Hayato Saigo[3], Kazuya Okamura[4], Hirokazu Hori[5], Aurélien Drezet[1],

Serge Huant[1], Guillaume Bachelier[1], Makoto Naruse[1,2]

[1] *Université Grenoble Alpes, CNRS, Institut Néel, 38000 Grenoble, France*

[2] *Department of Information Physics and Computing, Graduate School of Information Science and Technology, The University of Tokyo, 7-3-1 Hongo, Bunkyo-ku, Tokyo 113-8656, Japan*

[3] *Nagahama Institute of Bio-Science and Technology, 1266 Tamura, Nagahama, Shiga 526-0829, Japan*

[4] *Toyohashi University of Technology, 1-1 Hibarigaoka, Tempaku, Toyohashi, Aichi, 441-8580, Japan*

[5] *Interdisciplinary Graduate School, University of Yamanashi, Takeda, Kofu, Yamanashi 400-8510, Japan*

* nicolas_chauvet@ipc.i.u-tokyo.ac.jp

Section 1. Single-player and two-non-cooperative-player decision-making strategies

We describe the case when only Player 1 plays the slot machines. If in cycle t, the selected machine is Machine A and it yields a reward (in other words, if Player 1 wins by playing Machine A), then the polarization adjuster value of Player 1 (PA_1) is updated at cycle $t + 1$ according to

$$PA_1(t+1) = -\Delta_1 + \alpha_1 PA_1(t), \tag{S1}$$

where α_1 is a forgetting parameter[S1,S2] and Δ_1 is a constant increment. In this experiment, $\Delta_1 = 1$ and $\alpha_1 = 0.999$. The initial value of PA_1 is zero. If the selected machine (Machine A) does *not* yield a reward (i.e. if Player 1 loses), PA_1 is updated according to

$$PA_1(t+1) = +\Omega_1 + \alpha_1 PA_1(t), \tag{S2}$$

where Ω_1 is a parameter that is adaptively configured concerning the history[S2]. In this study, Ω_1 was a constant ($\Omega_1 = 1$), assuming the summation of the reward probabilities to be known ($P_A + P_B = 1$). Intuitively speaking, PA_1 *decreases* if Machine A is more likely to win and *increases* if Machine B is considered to be more likely to earn rewards. The value of PA_1 is then adapted to polarization control of HP_1. Specifically, the orientation of HP_1 at cycle t is determined by

$$HP_1(t) = \mathrm{POS}_1\left(\lceil PA_1(t) \rceil\right), \tag{S3}$$

where $\lceil\ \rceil$ represents the round-off function to the closest whole number. In the experiment, the rounded integer PA values were −3, −2, −1, 0, 1, 2, and 3. When $PA_1(t) \geq 4$ or $PA_1(t) \leq -4$, the rounded PA value was defined by $\lceil PA_1(t) \rceil = 3$ and $\lceil PA_1(t) \rceil = -3$, respectively. The actual values of the orientations set according to Eq. (S3) were determined by considering the asymmetry of the photon counts between APD1 and APD2, as discussed in the main article. Specifically, POS(–3), POS(–2), POS(–1), POS(0), POS(1), POS(2), and POS(3) were given by −18.7°, –15.3°, –11.3°, 0°, 11.8°, 16°, and 21.2°, which are marked by the dotted lines in Fig. S1a, so that the ratio of the photon count by

APD1 to that by APD2 was 20, 10, 5, 1, 1/5, 1/10, and 1/20, respectively, to resolve the asymmetry effectively. In addition, to prevent the PA value from being too large or too small, which could limit the speed of adaptation to environmental changes, the maximum and minimum PA values were set to 10 and −10, respectively.

The adaptive decision making of Player 2 was implemented in the same way. The orientation of HW_2 was tuned differently to account for the sensitivities of APD3 and APD4. Specifically, POS(−3), POS(−2), POS(−1), POS(0), POS(1), POS(2), and POS(3) were −19.2°, −17.1°, −13.6°, 0°, 15°, 19.6°, and 21.5°, which are marked by the dotted lines in Fig. S1b, so that the ratio of the photon count by APD3 to that by APD4 was 50, 30, 10, 1, 1/10, 1/30, and 1/50, respectively, to resolve the asymmetry effectively.

For a given slot machine play, the actual decision of Player 1 was made by the *identity* of the channel where the *first* photon arrival was measured[S3]. If the first photon was detected by APD1, the decision was to select Machine A, whereas if it was detected by APD2, the decision was to choose Machine B. Likewise, the decision of Player 2 was made based on the first photon detection either by APD3 or APD4.

Section 2. Implementation of collective decision making

Using the multiple-event time digitizer, four kinds of coincidence of observing photons at (i) APD1 and APD3, (ii) APD2 and APD3, (iii) APD1 and APD4, and (iv) APD2 and APD4 were measured. For a given slot machine play, the *identity* of the *first* observation of the coincidence among the four possible combinations was considered to represent the decisions of Players 1 and 2. The correspondence between the photon measurement and the decision to be made was the same as in the single-player cases described in Sec. 1; for example, measurement of a photon by APD1 corresponded to the decision of choosing Machine A for Player 1, while the detection of a photon by APD3 corresponded to the decision of choosing Machine A for Player 2. Therefore, for example, if the first

coincident observation was made by APD1 and APD3, the decision of Player 1 was to choose Machine A, while that of Player 2 was also to choose Machine A.

Fig. S1c characterizes the optical system by presenting the coincidence rate statistics measured over 2 s regarding the joined photodetection as a function of the rotation of HW_1 and HW_2. For that we used the following sets of detectors: (i) APD1 and APD3 (downward triangles), (ii) APD1 and APD4 (circles), (iii) APD2 and APD3 (diamonds), and (iv) APD2 and APD4 (upward triangles). The large coincidences of (ii) and (iii), and smaller coincidences of (i) and (iv) throughout the polarization basis clearly demonstrate the successful establishment of cross-polarized entangled photon pairs.

The total rewards accomplished by correlated and entangled photon pairs presented in Fig. 3 in the main manuscript are the average accumulated rewards at cycle 100 when the polarization bases were 0°, 15°, 30°, and 45°. This was done to ensure fair comparison between the correlated and entangled photons concerning the contrasting behavior in the case of the correlated photons with polarization bases of 0° and 45°.

Section 3. Definition of equality

The equality shown in Figs. 3a,iii and 3b,iii in the main manuscript was evaluated as follows. (1) Calculate the ratio between the average numbers of times that Players 1 and 2 selected the higher-reward-probability machine from the first play to the 50th play. (2) Calculate the ratio between the average numbers of times that Players 1 and 2 selected the higher-reward-probability machine from the 51st play to the 100th play. (3) Calculate the average of (1) and (2). This average is a reasonable metric regarding equality of the opportunities to select the higher-reward-probability machine taking into account the fact that the casino setting was different between the first 50 cycles and the next 50 cycles.

Section 4. Dependence of total rewards on casino setting

In order to check the sensitivity of the decision-making performance with respect to the reward probabilities P_A and P_B, they were changed to $P_A = 0.4$ and $P_B = 0.6$ from $P_A = 0.2$ and $P_B = 0.8$. It appeared that finding the higher-reward-probability machine was more difficult in that case due to the smaller difference between the reward probabilities than in the former cases. The blue bars in Fig. S2 show the accumulated rewards at cycle 100. Indeed, for Player 1 only, Player 2 only, and two non-cooperative players, the total reward is substantially lower than in the former cases (depicted by the red bars). These differences are due to the longer time needed to reach stable selection of the higher-reward-probability machine. On the contrary, with correlated and entangled photons, the team reward does not change, and the entangled photons again provide the maximum total reward. This finding clearly demonstrates that collective decision making based on entangled photons ensures that the social maximum reward will be achieved regardless of the difficulty of the given problem. This has strong implications in terms of resource allocation, for example in network communications, as the maximized efficiency is ensured whatever the actual qualities of the two channels, which may actually fluctuate in time.

Section 5. Randomly cross-polarized photon pairs

Polarization-correlated and polarization-entangled photon pairs show very different characteristics in the configuration used for these experiments, especially fixed polarization state for the former and coherent sum of states for the latter. In particular, a player can fully determine the polarization state of polarization-correlated photon pairs by using his/her half-waveplate, whereas it is impossible to do so for polarization-entangled photon pairs. Another kind of photonic state can be used to compare with entangled photon pairs: a series of cross-polarized photon pairs along random directions. In this situation, using the same notations as in the main text, each photon pair has a polarization state of the kind:

$$|\Psi\rangle = |\theta, \theta + \pi/2\rangle, \theta \in [0,2\pi[\ . \tag{S4}$$

With this input state $|\Psi\rangle$, players have 50% probability on average to select either machine A or machine B whatever the angle of their own waveplate: as a consequence, individual rewards are necessarily identical between players on average. The only way for players to change the total rewards is to tune the conflict rate by modifying the relative angle between their waveplates.

Simulations have been run to check this behavior with the following set-up: at each play, a uniformly distributed pseudorandom number is used to select the angle θ of the state $|\theta, \theta + \pi/2\rangle$ as input photon pair state for the players, with each photon then following the same rules and transformations as for polarization-correlated pairs. The reward probabilities follow the same rules as before, with $P_A = 0.2$ and $P_B = 0.8$.

Figure S3 shows numerical results with cooperative players (i.e., fixed waveplate angle) for **a** identical polarization bases, **b** polarization bases tilted by 45 degrees from each other and **c** polarization bases tilted by 90 degrees. As predicted, while the correct decision ratio (CDR) shown in item (i) is independent from the polarization bases (CDR stays around 0.5) and identical for both player on average, conflict ratio in (ii) varies according to the relative angle tilt. These changes explain the differences in accumulated reward in (iii) between the three cases, bases alignment leading to an average accumulated total reward of 87.9 in **a** whereas 50% conflict ratio lowers it to 74.7 in **b** and higher ratio in **c** lowers it to 62.7. These results indicate that the outcome depends on the relative angle between waveplates, as is the case for entangled photon pairs, though the maximum total accumulated reward of 100 cannot be reached because of residual conflicts due to experimental imperfections.

Figure S4 summarizes the comparison between the two kinds of resources and their performance with respect to misalignment angle between players' bases, by presenting the accumulated total reward for different measurement bases tilts between players. In particular, if entangled photon pairs enable players to reach maximum accumulated reward when their bases are mutually aligned, they suffer heavier losses when the misalignment is higher than 45 degrees as

compared with randomly cross-polarized photon pairs. The lower sensitivity obtained with this kind of resource may then be of interest for applications more susceptible to noise and perturbations between players.

References

S1. Naruse, M. *et al.* Single-photon decision maker. *Sci. Rep.* **5,** 13253 (2015).

S2. Naruse, M. *et al.* Single Photon in Hierarchical Architecture for Physical Decision Making: Photon Intelligence. *ACS Photonics.* **3,** 2505–2514 (2016).

S3. Fedrizzi, A., Herbst, T., Poppe, A., Jennewein, T., Zeilinger, A. A wavelength-tunable fiber-coupled source of narrowband entangled photons. *Opt. Express* **15,** 15377–15386 (2007).

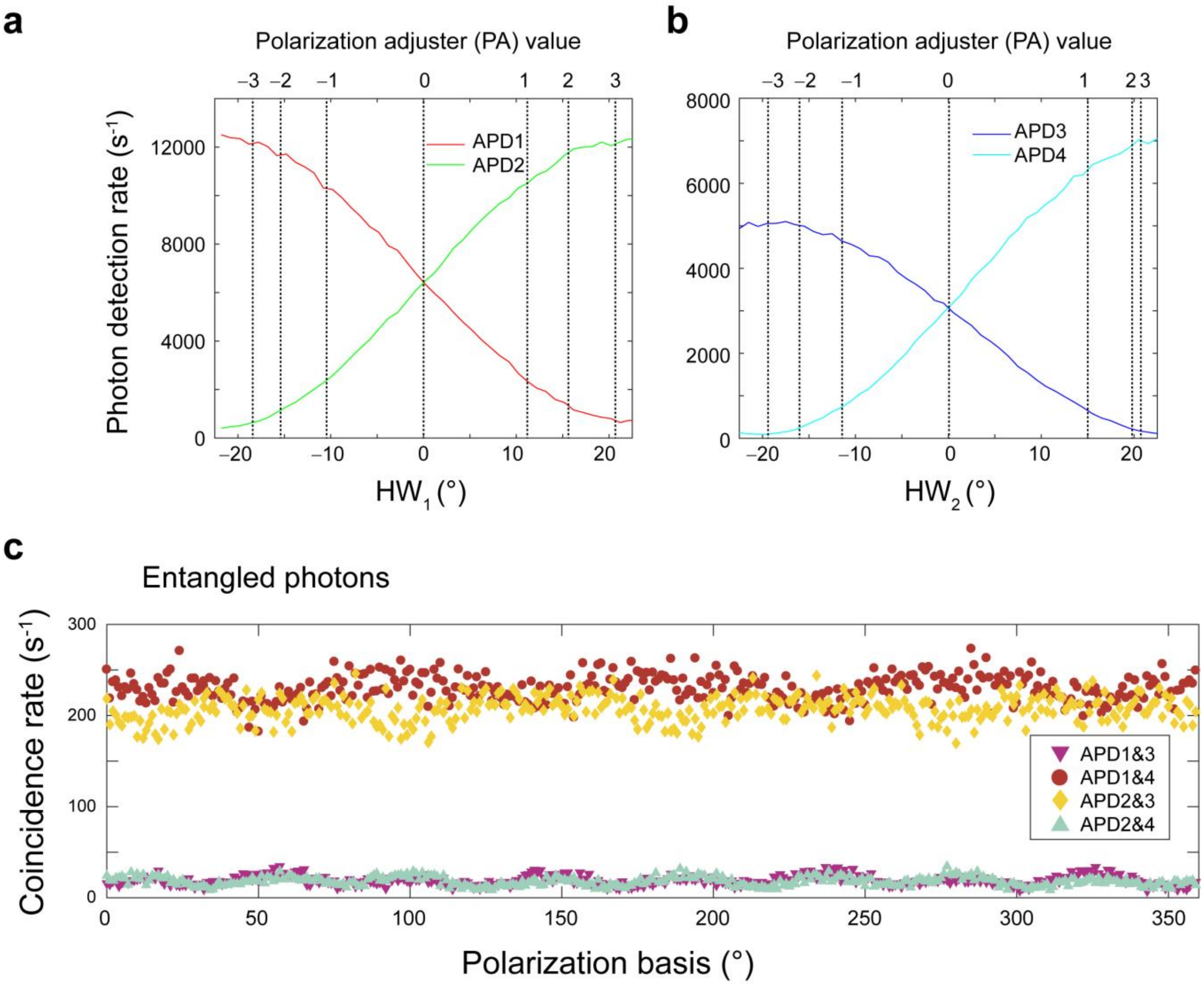

Fig. S1 | Characterization of the optical system. a With *non*-entangled photon generation and with the insertion of polarizers, the photon statistics related to Player 1 (APD1 and APD2) behave symmetrically (although not perfectly due to experimental imperfections) depending on the orientation of HW_1, which is located in front of PBS_1. **b** Similarly, the photon statistics related to Player 2 (APD3 and APD4) behave symmetrically depending on the orientation of HW_2, which is located in front of PBS_2. **c** Photon pair statistics when *entangled* photons are generated. Regardless of the common wave plate orientations (HW_1 and HW_2), consistent coincidence rates are observed along the polarization bases.

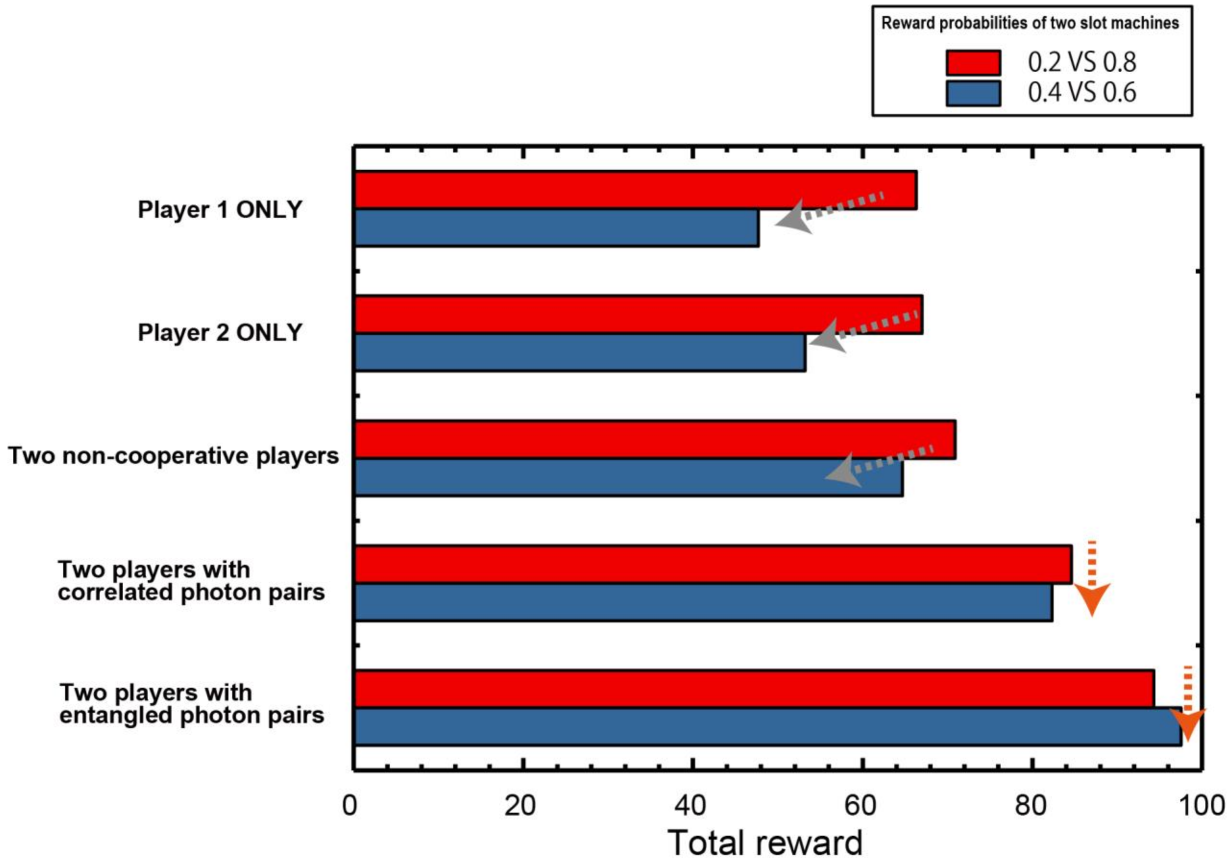

Fig. S2 | Dependence of total rewards to casino setting. As the difficulty of the given problem increases (from 0.2 VS 0.8 to 0.4 VS 0.6), the total rewards in the cases of single players and two non-cooperative players decrease whereas those in the cases of correlated and entangled photon pairs remain the same. The entangled photons provide the maximum rewards.

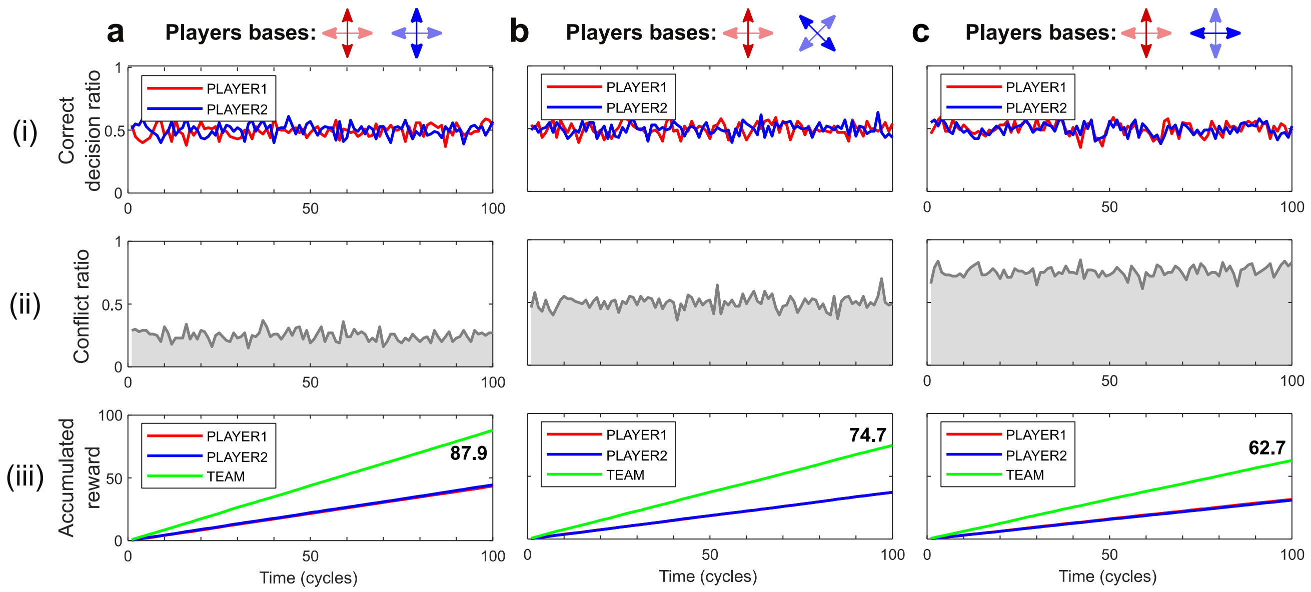

Fig. S3 | Randomly cross-polarized photon pairs with cooperative players. Cross-polarized photon pairs with random orientation show different behaviour with respect to polarization-entangled photon pairs, even if measurement bases are the same between players. **a** If polarization bases are aligned, total accumulated reward of 87.9 is obtained and equally shared between players, which is 12% below simulations for entangled photon pairs because of non-zero conflict ratio. **b** If bases are tilted by 45 degrees, this ratio increases to 50% and reward decreases to 74.7, while **c** bases tilted by 90 degrees show the worse outcome with 62.7 accumulated total reward.

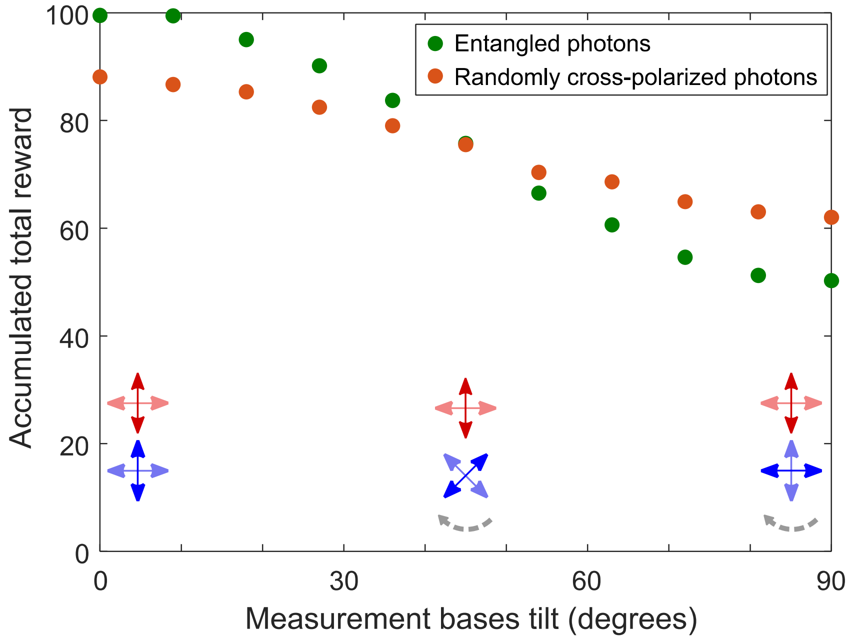

Fig. S4 | Randomly cross-polarized vs entangled photon pairs with cooperative players. Comparison of the simulated accumulated total reward between entangled photons (green) and randomly cross-polarized photon pairs used by cooperative players (fixed waveplate position), with respect to the angle difference between players' bases. With perfectly aligned polarization bases, entangled photons enable players to reach nearly maximum reward (99.5 here), while cross-polarized photon pairs only reach 88.1. However, misalignment between bases has a greater impact with entangled photons, with the case of 90 degrees tilt showing 50.3 accumulated total reward versus 62 for cross-polarized photons.
